# Maternal nutritional adequacy and gestational weight gain and their associations with birth outcomes among Vietnamese women

**DOI:** 10.1186/s12884-019-2643-6

**Published:** 2019-12-04

**Authors:** Nga Thuy Tran, Lam Thi Nguyen, Yatin Berde, Yen Ling Low, Siew Ling Tey, Dieu Thi Thu Huynh

**Affiliations:** 1 Micronutrient Research and Application, National Institution of Nutrition, 48B Tang Ba Ho, Hai Ba Trung District, Ha Noi, Vietnam; 2 Clinical Nutrition, National Institution of Nutrition, 48B Tang Ba Ho, Hai Ba Trung District, Ha Noi, Vietnam; 3 Statistical Services, Cognizant Technologies Solution Pvt. Ltd, Hiranandani Business Park, Mumbai, 400076 India; 4 Abbott Nutrition Research and Development Asia-Pacific Center, 20 Biopolis Way, Unit 09-01/02 Centros Building, Singapore, 138668 Singapore

**Keywords:** Maternal nutritional status, Gestational weight gain, Nutritional adequacy, Birth outcomes

## Abstract

**Background:**

During pregnancy, a mother’s nutritional needs increase to meet the added nutrient demands for fetal growth and development. An enhanced understanding of adequate nutrition and sufficient weight gain during pregnancy can guide development of policies and strategies for maternal nutrition care, actions that will ultimately promote better pregnancy outcomes. In a sample of pregnant women in Vietnam, this study characterized maternal nutrition status and gestational weight gain at a mid-pregnancy baseline, then examined the association of these variables with specific birth outcomes.

**Methods:**

The study used baseline data from a randomized, controlled trial that compared pregnant Vietnamese women who received a nutritional intervention group with those who received only standard dietary counseling (control group). At baseline (26–29 weeks gestation), mothers’ dietary reports were collected, and intake of 10 macro- and micronutrients was estimated; data for baseline gestational weight gain was collected for all pregnant women enrolled into the study (*n* = 228). This analysis also used weights, lengths, and head circumferences at birth for infants of mothers in the control group.

**Results:**

At baseline, 95% of the pregnant women had concurrent inadequacies for more than five nutrients, and nearly half had concurrent inadequacies for more than ten nutrients. Almost two-thirds of the pregnant women did not meet recommendations for gestational weight gain. We found a significant, inverse association between the number of nutrient inadequacies and gestational weight gain (overall *p* ≤ 0.045). After adjusting for potential confounders, gestational weight gain was positively associated with birth weight, length at birth, birth weight-for-age *z*-score and length-for-age *z*-score (all *p* ≤ 0.006).

**Conclusions:**

Our findings raise concern over the high proportion of pregnant women in Vietnam who have multiple concurrent nutrient inadequacies and who fall short of meeting recommended gestational weight gain standards. To ensure better birth outcomes in this population, policies and strategies to improve the status of maternal nutrition are greatly needed.

**Trial registration:**

The trial was retrospectively registered at clinicaltrials.gov on December 20, 2013, registration identifier: NCT02016586.

## Background

Pregnancy is a crucial time for women to be well-nourished. The added nutrient demands of fetal growth and development must be met in order to ensure optimal birth and growth outcomes. A suboptimal maternal diet and inadequate gestational weight gain during pregnancy increase risk for adverse health outcomes for both mother and child [1–5].

Inadequate macronutrient intake is common among pregnant women in some Asian countries, and certain micronutrient deficiencies are also frequent. Nutrients of particular concern include iron, zinc, folate, vitamin A, vitamin D, iodine, and calcium, which play significant roles in maternal health and fetal development [6–11]. Nutrient inadequacies during pregnancy can impair fetal growth, which can in turn increase risk for low weight at birth or small-for-gestational-age and preterm deliveries [12–16]. Inadequate nutrient intakes during this time may also lead to reprogramming within fetal tissues, which is associated with increased risk for non-communicable chronic diseases in adulthood [5, 17–20]. Thus, for pregnant women who have difficulty achieving recommended nutrient intake through usual diet alone, it is strategic to intervene with supplements containing protein, energy, and other nutrients.

Suboptimal weight gain during pregnancy has also been reported as highly prevalent in studies of other Asian populations [21–24]. Results of such studies found significant associations between inadequate gestational weight gain and poor pregnancy outcomes [21–30]. Findings from a recent meta-analysis showed that inadequate gestational weight gain was associated with increased risk of preterm birth by 70% (95% CI: 32 to 120%) and increased risk of delivering a small-for-gestational-age infant by 53% (95% CI: 44 to 64%) [26]. Further, such small infants may experience nutritional shortfalls owing to failure to initiate breastfeeding. Dietary inadequacy in infants can lead to impaired growth and cognitive development in childhood, and even to adverse metabolic consequences in adulthood [31–33]. Strategies aimed to promote appropriate weight gain during pregnancy have the potential to improve birth outcomes, and these strategies may also generate significant intergenerational benefits [32, 34–37].

To our knowledge, data are limited on maternal nutritional status during pregnancy and its association with birth outcomes in developing countries such as Vietnam. The objectives of the present study were to (1) report adequacy of maternal nutrition and gestational weight gain at baseline (26–29 weeks gestation) among all the pregnant women enrolled in this Vietnamese study, and to (2) examine the association of these factors with specific birth outcomes (infant weight, length, and head circumference or the related z-scores for each) for infant’s whose mothers were in the standard care (control) group.

Better understanding of associations between markers of maternal nutrition status and infant birth outcomes can guide development of appropriate prenatal nutrition policies, guidelines, and practices for better outcomes to pregnancy.

## Methods

### Study design and population

The present study analysed a subset of data collected in a prior study, which has been described previously [38]. Briefly, the full study was a prospective, randomized, open-label, parallel-group, multi-center design. It was conducted in 20 community medical stations and district hospitals across four Northern provinces in Vietnam between October 2013 and April 2015. Study eligibility requirements for women were: healthy and pregnant, 20–35 years of age, first-time mothers with singleton pregnancies, at 26 to 29 weeks of gestation, and with pre-pregnancy body mass index (BMI) < 25.0 kg/m^2^ (not overweight or obese).

For the original study, a total of 228 singleton mothers took part in a randomized controlled trial with two groups: *Intervention -* daily maternal nutritional supplementation containing macronutrients and a variety of micronutrients starting from baseline (26–29 weeks gestation) to 12 weeks postpartum plus a breastfeeding support program (intervention group) or (B) *Control -* standard-of-care treatment, including iron and folic acid supplementation until delivery. For the present analysis, we included data on mothers’ socio-demographic characteristics, nutritional adequacy, and gestational weight gain at baseline; we included data on infants’ weights, lengths, and head circumferences at birth.

### Maternal characteristics and nutritional status

Descriptive maternal characteristics were collected at baseline. Socio-demographics included questions on maternal age, and highest education qualification. Economic status was determined by constructing a wealth index based on asset ownership from an inventory of household assets [39].

Maternal nutritional status was assessed using anthropometric measurements and dietary intake. Self-reported pre-pregnancy weight or the earliest measured weight in the first trimester from hospital record was recorded. In addition, measured height, weight, and mid-upper arm circumference were collected at baseline. Standing height and weight were measured using a height and weight scale (Horse Head Brand TZ-120). Body mass index (kg/m^2^) was calculated by dividing weight (kg) by height squared (m^2^) and maternal underweight was defined as having a pre-pregnancy BMI < 18.5 kg/m^2^ [40, 41]. Gestational weight gain during the first and second trimesters was calculated by subtracting pre-pregnancy weight from weight at 26 to 29 weeks and this was compared with the Institute of Medicine (IOM) recommended gestational weight gain to determine whether the pregnant women met the IOM recommendation based on their pre-pregnant BMI and the recommended weight gain at the particular trimester of their pregnancy [40]. Mid-upper arm circumference was measured from the upper left arm using a measuring tape and a cut-off of < 23.0 cm was considered suboptimal nutritional status [42]. Dietary intake was collected at baseline by trained research staff using a standardized 24-h recall method. Nutrient adequacy was defined as 77% of the 2016 recommended daily allowance (RDA) for Vietnam [43] as the cutoff value [44]. We chose this criterion and cutoff because the estimated average requirement (EAR) is available for some (but not all) nutrients in Vietnam.

### Birth outcomes

Medical records were used to obtain information on delivery mode (vaginal or caesarean), as well as gestational age, infant’s weight, length, and head circumference at birth. The weight and length of the infant were obtained using a Seca 232 measuring rod. The head circumference was determined by measuring tape. World Health Organization (WHO) Child Growth Standards were used to calculate the sex-age-specific *z*-scores for infant’s weight, length, and head circumference [45]. Small-for-gestational-age was defined as birth weight and/or birth length at least two standard deviations below the mean level for sex and gestational age [46].

### Statistical analysis

Maternal characteristics and nutrient inadequacies of all participants (*n* = 226) were presented as arithmetic means and standard deviations for continuous variables; categorical data were presented as numbers and percentages.

A simple linear regression model was used to examine the association at baseline of each single nutrient and as concurrent nutrient inadequacies (grouped as ≤5, 6–10, and 11–15 inadequate nutrients) with gestational weight gain at 26 to 29 weeks in all participants (*n* = 226).

Multiple regression analyses were used to assess the association of various factors with birth outcomes (birth weight, length, head circumference, birth weight-for-age *z*-score, length-for-age *z*-score, and head circumference-for-age *z*-score) in the control group only (*n* = 113). These factors (at baseline) included mother’s age, educational level, economic status, gestational weight gain, nutrient inadequacies, energy intake, energy-adjusted macronutrient intake, and infant’s gestational age at birth and sex.

The modelling process for multiple regression analyses was started with the bivariate analysis in which the association between a single factor and the birth outcome was examined. Factors with *p*-values < 0.2 from preliminary models were included in the final model. Mother’s age, gestational age of infant at birth, and infant’s sex are known to influence birth outcomes, so these three variables were also included in the final multiple regression models. The adjusted estimates and the 95% confidence interval derived from the final models were reported. All variables that were included in the models were defined a priori*,* and collinearity was examined in all the models. Data from the original intervention group were not included in any of the analyses for birth outcomes, as those mothers received maternal nutritional supplementation and breastfeeding support during the study, which significantly improved birth outcomes [38].

Birth outcomes were compared between study groups using analysis of covariance (ANCOVA) to control for confounding factors, including treatment group, mother’s age, mother’s nutritional status at baseline, socioeconomic status, gestational age and infant’s gender.

SAS version 9.3 (SAS Institute, Cary, NC, USA) was used for all statistical analyses. All statistical tests were two-sided and *p* < 0.05 was considered statistically significant.

## Results

### Maternal characteristics

Of the 228 mothers enrolled into the study, 226 women were successfully randomized to either the intervention group (*n* = 113) or the control group (*n* = 113) and were included in an intention-to-treat analysis [38]. As shown in Table 1, the mean (±SD) age of the mothers was 24.0 (±2.9) years old, and the mean (SD) self-reported pre-pregnancy BMI was 19.2 (±1.8) kg/m^2^, increasing to 22.2 (±2.2) kg/m^2^ by 26 to 29 weeks of gestation. Approximately one-third of the women (32.7%) were underweight, defined as pre-pregnancy BMI < 18.5 kg/m^2^ prior to pregnancy and 4.0% of them remained underweight at 26 to 29 weeks of gestation. Almost two-thirds of the women did not meet the gestational weight gain recommended by the IOM at the mid-pregnancy interval of 26 to 29 weeks gestation. At this time, 29.2% of the mothers also had suboptimal nutritional status, as defined by mid-upper arm circumference < 23.0 cm.

**Table 1:** Baseline characteristics of all mothers (*n* = 226)^a^

Baseline characteristics	Mean (±SD) / n (%)
Age (years), mean (SD)	24.0 (2.9)
Economic status, *n* (%)	
Low	72 (31.9)
Medium	83 (36.7)
High	71 (31.4)
Highest education qualification, *n* (%)	
Primary school	3 (1.3)
Secondary school	59 (26.1)
High school	79 (35.0)
College / University	85 (37.6)
Height (cm), mean (SD)	154 (5.0)
Pre-pregnancy weight (kg), mean (SD)	45.5 (5.1)
Pre-pregnancy BMI (kg/m^2^), mean (SD)	19.2 (1.8)
Pre-pregnancy BMI (kg/m^2^), *n* (%)	
< 18.5	74 (32.7)
≥ 18.5	152 (67.3)
Weight at 26 to 29 weeks gestation (kg), mean (SD)	52.7 (5.9)
BMI at 26 to 29 weeks gestation (kg/m^2^), mean (SD)	22.2 (2.2)
BMI at 26 to 29 weeks gestation (kg/m^2^), *n* (%)	
< 18.5	9 (4.0)
≥ 18.5	217 (96.0)
Gestational weight gain at 26 to 29 weeks gestation (kg), mean (SD)^b^	7.1 (2.7)
Met IOM recommended gestational weight gain at 26 to 29 weeks gestation (kg/m^2^), *n* (%)	
Yes	85 (38.5)
No	136 (61.5)
MUAC at 26 to 29 weeks gestation (cm), mean (SD)	24.2 (2.2)
MUAC at 26 to 29 weeks gestation (cm), *n* (%)	
< 23.0	66 (29.2)
≥ 23.0	160 (70.8)
Nutrient inadequacies, *n* (%)^c^	
≤ 5 nutrients	12 (5.3)
6–10 nutrients	114 (50.4)
11–15 nutrients	100 (44.3)

*BMI* body mass index, *MUAC* mid-upper arm circumference

^a^Data are presented as means (standard deviations) or numbers (percentages)

^b^Gestational weight gain at 26–29 weeks is calculated by subtracting pre-pregnancy weight from measured weight at 26–29 weeks

^c^Selected nutrients include protein, calcium, iron, zinc, vitamins A, D, E, C, B1, B2, B3, B5, B6, B9, and B12

#### Nutrient inadequacy at 26 to 29 weeks of gestation

At the baseline data collection (26–29 weeks gestation), 95% of all the pregnant women in this study (*n* = 226) had concurrent inadequacies for more than five nutrients, and approximately one in two (44%) had inadequacies for more than ten nutrients (Table 1). The fifteen nutrients that were included in this analysis were protein, calcium, iron, zinc, vitamins A, D, E, C, B1, B2, B3, B5, B6, B9, and B12 (Table 1). Mean (SD) energy and nutrient intakes (without prenatal supplements) at 26 to 29 weeks of gestation in all study participants can be found in Table 2.

**Table 2:** Nutrient intake at 26 to 29 weeks of gestation in all study participants (*n* = 226)^a^

Variables	Mean (SD)
Energy (kcal)	2065 (388)
Protein (g)	81.4 (17.0)
Fat (g)	45.2 (16.5)
Carbohydrate (g)	333 (76.3)
Calcium (mg)	592 (286)
Iron (mg)	14.3 (5.4)
Zinc (mg)	11.4 (2.8)
Vitamin A (mcg)	608 (629)
Vitamin D (mcg)	1.23 (2.50)
Vitamin E (mg)	2.56 (1.83)
Vitamin C (mg)	144 (89.5)
Vitamin B1 (mg)	1.43 (0.49)
Vitamin B2 (mg)	0.95 (0.39)
Vitamin B3 (mg)	14.9 (5.7)
Vitamin B5 (mg)	6.56 (1.81)
Vitamin B6 (mg)	1.43 (0.39)
Vitamin B9 (mcg)	269 (162)
Vitamin B12 (mcg)	2.28 (3.05)

^a^Data are presented as means (standard deviations)

**Fig. 1:**
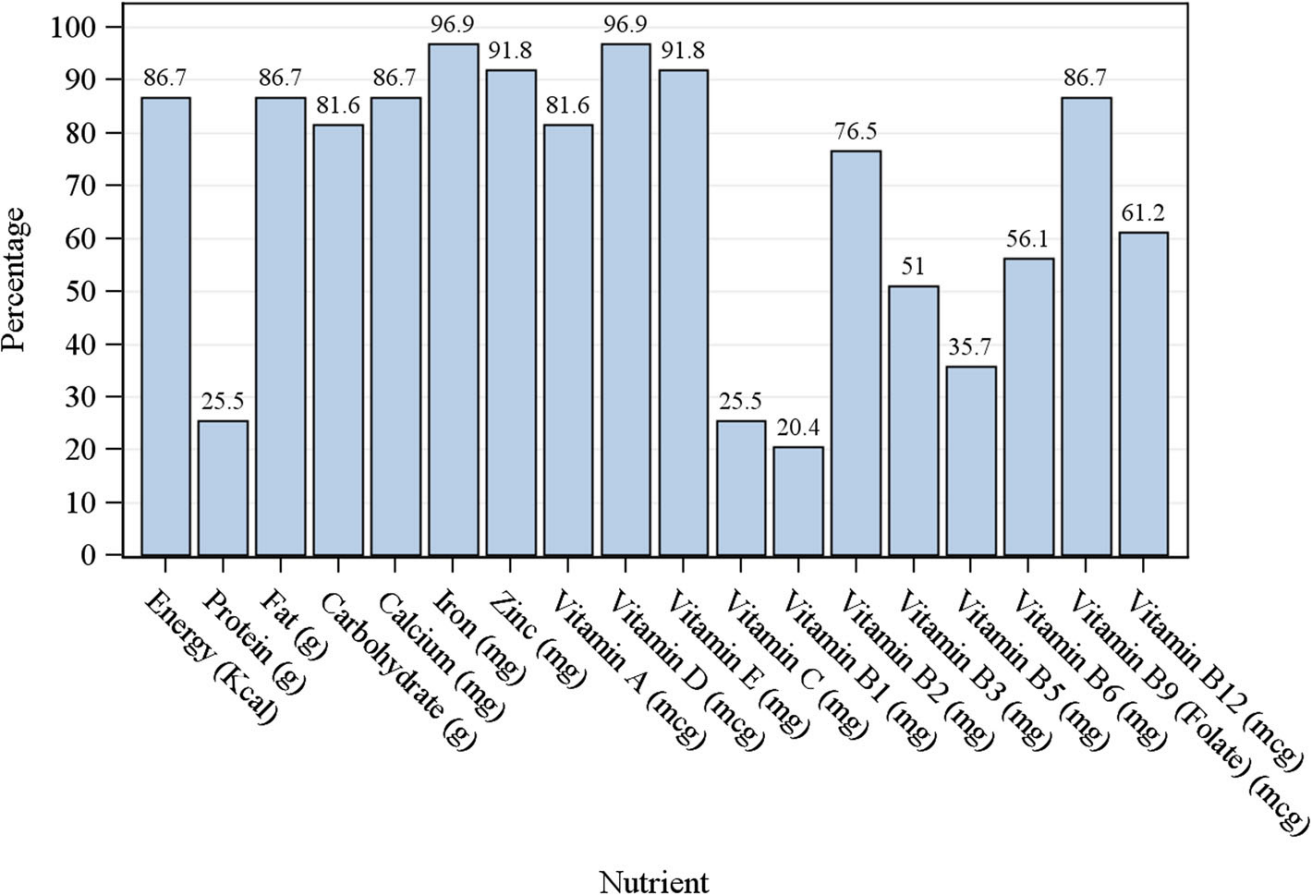
Percentage of pregnant women with inadequate intake (< 77% RDA) for selected nutrients at 26 to 29 weeks gestation in all participants (*n* = 226)

Figure 1 shows the percentage of pregnant women with inadequate nutrient intake at baseline (26 to 29 weeks gestation). Over three-quarters of the pregnant women did not meet the adequate intakes for energy, fat, carbohydrate, calcium, iron, zinc, vitamins A, D, E, B2, and folate at baseline.

There was a significant inverse relationship between the number of nutrient inadequacies (≤5, 6–10, 11–15) and gestational weight gain at 26 to 29 weeks (overall *p* = 0.045; data not shown). Pairwise comparisons showed that mothers who had 6–10 nutrient inadequacies had lower gestational weight gain at 26 to 29 weeks than did those with ≤5 nutrient inadequacies (mean difference = − 1.05 kg, 95% CI: − 2.63, 0.53; *p* = 0.192), although this difference did not reach statistical significance. Mothers who had 11–15 nutrient inadequacies had significantly lower gestational weight gain at 26 to 29 weeks than mothers who had ≤5 nutrient inadequacies (mean difference = − 1.71 kg, 95% CI: − 0.11, − 3.30; *p* = 0.036). Gestational weight gain was positively correlated with the intakes of energy, carbohydrate, protein, calcium, iron, vitamin C, vitamin B2, vitamin B5, and vitamin B6 (all *p* ≤ 0.030) in univariate analyses (data not shown).

### Birth outcomes by study groups

The mean (SD) gestational age at birth for control and intervention group was 39.1 (±1.6) weeks and 39.1 (±1.3) weeks, respectively, infant’s birth weight was 3044 (±385) g and 3153 (±347) g, respectively, birth length was 48.7 (2.25) cm and 49.0 (±1.7) cm, respectively, and birth head circumference was 32.8 (±1.64) cm and 33.1 (±1.3) cm, respectively (Table 3). The infants in the intervention group had significantly higher birth weight than did those in the control group (*p* = 0.0346). There were no significant differences in birth length and birth head circumference between the two groups.

**Table 3:** Birth outcomes by study group

Characteristics	Intervention *N* = 113	Control *N* = 113	*p*-value
Gestational age, mean (SD)	39.1 (1.3)	39.1 (1.6)	0.8026^a^
Delivery method, *n* (%)			
Caesarean	35 (31.5)	30 (27.3)	0.4872
Infant’s sex, n (%)			0.9503^b^
Male	60 (54.1)	59 (53.6)	
Female	51 (46.0)	51 (46.4)	
Birth weight (g), mean (SD)	3153 (347)	3044 (385)	0.0346^a^
Birth length (cm), mean (SD)	49.0 (1.7)	48.7 (2.2)	0.5195^a^
Head circumference (cm), mean (SD)	33.1 (1.3)	32.7(1.6)	0.1204^a^

^a^*p*-value is from Wilcoxon test, ^b^*p*-value is from T-test

### Factors associated with birth outcomes in the control group

Table 4 shows the factors that are associated with birth weight, length, and head circumference, as well as birth weight-for-age *z*-score, length-for-age *z*-score, and head circumference-for-age *z*-score in the control group only from the multiple linear analysis (*n* = 110). After adjusting for mother’s age at enrollment, gestational age at birth, and infant’s gender, gestational weight gain at 26 to 29 weeks was strongly associated with birth weight (adjusted estimate = 42.9 g; 95% CI: 17.5, 68.3), birth length (adjusted estimate = 0.23 cm; 95% CI: 0.07, 0.39), birth weight-for-age *z*-score (adjusted estimate = 0.10; 95% CI: 0.04, 0.15), and birth length-for-age *z*-score (adjusted estimate = 0.12; 95% CI: 0.04, 0.21) (all *p* ≤ 0.006). Similarly, education level was positively associated with birth weight (*p* = 0.029), head circumference (*p* = 0.046), birth weight-for-age *z*-score (*p* = 0.008), and a tendency was found for birth length (*p* = 0.072), birth length-for-age *z*-score (*p* = 0.076), and birth head circumference-for-age *z*-score (*p* = 0.061).

**Table 4:** Factors associated with birth outcomes in the control group (*n* = 110)^a^

	Adjusted Estimate (95% CI)	*P* value
Birth weight		
Mother’s age at enrollment	−3.9 (−24.5, 16.7)	0.706
Gestational age at birth	97.4 (58.4, 136)	< 0.001
Infant’s gender		0.329
Female (reference group)	0	
Male	60.4 (−61.7, 183)	
Education level		0.029
Primary school (reference group)	0	
Secondary school	687 (212, 1163)	
High school	729 (253, 1206)	
College / University	739 (248, 1229)	
Economic status		0.965
Low (reference group)	0	
Medium	12.7 (− 142, 167)	
High	25.6 (− 166, 217)	
Energy intake at baseline	0.04 (− 0.14, 0.22)	0.649
Energy adjusted carbohydrate intake	0.47 (−1.16, 2.09)	0.569
Gestational weight gain at 26 to 29 weeks	42.9 (17.5, 68.3)	0.001
Birth length		
Mother’s age at enrollment	0.04 (−0.09, 0.17)	0.538
Gestational age at birth	0.50 (0.24, 0.75)	< 0.001
Infant’s gender		0.665
Female (reference group)	0	
Male	−0.17 (− 0.94, 0.60)	
Education level		0.072
Primary school (reference group)	0	
Secondary school	3.41 (0.41, 6.41)	
High school	3.77 (0.78, 6.77)	
College / University	3.17 (0.15, 6.19)	
Gestational weight gain at 26 to 29 weeks	0.23 (0.07, 0.39)	0.006
Birth head circumference		
Mother’s age at enrollment	0.002 (−0.10, 0.10)	0.958
Gestational age at birth	0.34 (0.15, 0.54)	< 0.001
Infant’s gender		0.611
Female (reference group)	0	
Male	0.15 (− 0.43, 0.72)	
Education level		0.046
Primary school (reference group)		
Secondary school	2.73 (0.49, 4.98)	
High school	3.16 (0.92, 5.39)	
College / University	2.93 (0.65, 5.21)	
Economic status		0.711
Low (reference group)	0	
Medium	−0.14 (−0.87, 0.59)	
High	0.20 (− 0.70, 1.10)	
Gestational weight gain at 26 to 29 weeks	0.07 (−0.06, 0.19)	0.292
Birth weight-for-age *z*-score		
Mother’s age at enrollment	−0.01 (− 0.06, 0.03)	0.592
Gestational age at birth	0.22 (0.13, 0.30)	< 0.001
Infant’s gender		0.502
Female (reference group)	0	
Male	−0.09 (− 0.36, 0.18)	
Education level		0.008
Primary school (reference group)	0	
Secondary school	1.76 (0.70, 2.82)	
High school	1.88 (0.82, 2.94)	
College / University	1.91 (0.82, 3.01)	
Economic status		0.999
Low (reference group)		
Medium	0.0001 (−0.34, 0.34)	
High	0.01 (−0.42, 0.44)	
Energy intake at baseline	0.0001 (−0.0003, 0.0005)	0.619
Energy adjusted fat intake	−0.005 (− 0.015, 0.005)	0.344
Gestational weight gain at 26 to 29 weeks	0.10 (0.04, 0.15)	0.001
Birth length-for-age *z*-score		
Mother’s age at enrollment	0.02 (−0.05, 0.09)	0.541
Gestational age at birth	0.27 (0.13, 0.40)	< 0.001
Infant’s gender		0.024
Female (reference group)	0	
Male	−0.48 (−0.89, − 0.06)	
Education level		0.076
Primary school (reference group)	0	
Secondary school	1.79 (0.19, 3.39)	
High school	1.99 (0.39, 3.58)	
College / University	1.67 (0.06, 3.27)	
Gestational weight gain at 26 to 29 weeks	0.12 (0.04, 0.21)	0.006
Birth head circumference-for-age *z*-score		
Mother’s age at enrollment	0.002 (−0.08, 0.08)	0.965
Gestational age at birth	0.28 (0.13, 0.44)	< 0.001
Infant’s gender		0.251
Female (reference group)	0	
Male	−0.28 (−0.74, 0.20)	
Education level		0.061
Primary school (reference group)	0	
Secondary school	2.12 (0.29, 3.95)	
High school	2.45 (0.64, 4.27)	
College / University	2.28 (0.43, 4.14)	
Economic status		0.741
Low (reference group)	0	
Medium	−0.10 (−0.69, 0.50)	
High	0.16 (− 0.57, 0.89)	
Gestational weight gain at 26 to 29 weeks	0.05 (−0.05, 0.15)	0.288

^a^Results were derived from multiple linear regression models. Variables with *P* < 0.20 in the bivariate analysis as well as mother’s age, gestational age at birth, and infant’s sex were included in the multiple linear regression models

### Comparison of birth outcomes between study groups

We also included some previously published results comparing the birth outcomes between the control and the intervention groups from the original randomized controlled trial for easy reference; that trial evaluated the effects of an improved perinatal nutrition care regimen on birth outcomes [38]. Birth outcomes were compared between study groups using analysis of covariance (ANCOVA) to control for confounding factors, including treatment group, mother’s age, mother’s nutritional status at baseline, socioeconomic status, gestational age and infant’s gender. The infants in the intervention had significantly higher birth weight (*p* = 0.0312), birth weight-for-age z-score (*p* = 0.0141), and head circumference-for-age z-score (*p* = 0.0487), compared with the control (Table 5).

**Table 5:** Comparison of birth outcomes between study groups

Growth parameters	Estimate (95% CI)		*p*-value
	Intervention (*n* = 104)	Control (*n* = 100)	
Birth weight (g)	92 (8176)	Reference	0.0312^a^
Birth length (cm)	0.1 (− 0.3, 0.6)	Reference	0.5452 ^a^
Birth head circumference (cm)	0.3 (0.0, 0.6)	Reference	0.0886 ^a^
Birth weight-for-age z-score	0.25 (0.05, 0.45)	Reference	0.0141^b^
Birth length-for-age z-score	0.18 (−0.10, 0.46)	Reference	0.2116^b^
Birth head circumference-for-age z-score	0.31 (0.00, 0.62)	Reference	0.0487^b^

^a^*p*-value is from ANCOVA analysis controlling for treatment group, gestational age and infant sex

^b^ *p*-value from ANCOVA analysis controlling for treatment group, mother’s age, mother’s nutritional status at baseline, socioeconomic status, gestational age and infant’s sex

## Discussion

In this study, we examined the nutritional status of pregnant women in Vietnam, particularly the association of maternal nutrition status variables with birth outcomes. Our findings showed striking results—one in three women was underweight prior to pregnancy, and one in two pregnant women had concurrent inadequacies for more than ten nutrients at 26 to 29 weeks of gestation. Results also indicated that approximately two-thirds of the pregnant women did not meet the recommended gestational weight gain, as advised by the IOM. There was a proportional relationship between nutrient inadequacies and gestational weight gain, i.e., a higher number of nutrient inadequacies was associated with lower gestational weight gain at 26 to 29 weeks. Gestational weight gain at 26 to 29 weeks was strongly positively associated with birth weight and length in the control group after adjusting for potential confounders.

Our study findings are consistent with previous studies, which reported a prevalence of underweight ranging from 20 to 40% among women of reproductive age in Asian countries such as China [23], Japan [27] and Vietnam [24, 25, 29, 47–50]. Two recent meta-analyses reported that maternal underweight was significantly associated with increased risks for preterm birth by 13 to 30%, having an infant with low birth weight by 66 to 67%, and small for gestational age by 67 to 85% [41, 51]. Similar findings had been reported in several other reviews, and these findings could be in part due to fetal growth restriction as a result of nutrient inadequacies [29, 30, 32, 52, 53].

We are concerned that a large proportion of pregnant women in the present study did not have adequate intakes for a number of key nutrients that are critical to maternal and infant health—calcium, iron, zinc, vitamins A, Bs, D, and E. These observations are consistent with previous studies reporting high prevalence of micronutrient deficiencies [47, 48, 54] and insufficient intakes of iron, zinc, folate, vitamin B12, and vitamin A [48, 55] among women of reproductive age or those who are pregnant in Vietnam, particularly among those living in suburban or with lower socio-economic status. When the results are taken together, it appears that the diet for the majority of Vietnamese women falls short of many essential nutrients and multiple nutrient inadequacies often coexist. This observation highlights the importance of improving the intake of a significant number of nutrients in this population group, in addition to the routine iron and folic acid supplementation as part of the standard care. Nutrient requirements increase significantly during pregnancy to support fetal growth and development. Inadequate nutrient intakes during this time may lead to reprogramming within fetal tissues, which could potentially increase the infant’s risk of non-communicable chronic diseases in adulthood [5, 17–20]. Recent evidence has shown that dietary interventions during pregnancy significantly improved birth outcomes, especially among women who were malnourished during pregnancy or who had a suboptimal diet [1, 2, 12, 15, 16, 56, 57].

Suboptimal weight gain during pregnancy and low birth weight can have long-lasting effects on the child. The present study results reinforce the positive association between gestational weight gain at 26 to 29 weeks and infant’s birth weight and length. Numerous studies have consistently demonstrated the efficacy of dietary interventions during pregnancy in lowering the incidence of preterm birth, low birth weight, and small for gestational age and this effect appears to be more pronounced among women who are undernourished during pregnancy [1, 2, 4, 36, 57]. In addition, factors such as poor maternal nutritional status before and during pregnancy, infant’s low birth weight and small for gestational age, have been suggested to adversely affect the growth and cognitive development of the offspring [3, 58, 59], and any increase in birth weight within the normal range has the potential to improve these outcomes after adjusting potential confounders. Therefore, the provision of energy, macronutrient, and micronutrient intakes such as maternal nutritional supplementation to the mothers who have inadequate gestational weight gain during the first and second trimesters could be an effective way to meet nutrient needs and to optimize birth outcomes [1, 15, 30, 34, 53, 57]. Indeed, the present study showed that maternal nutritional supplementation in conjunction with breastfeeding support significantly improved infant’s birth weight, weight-for-age *z*-score, and head circumference-for-age *z*-score compared to the control group (all *P* ≤ 0.049) [38].

The strength of the current study is that it includes the measure and reports on a comprehensive range of demographic, anthropometric, and dietary variables, which advances our understanding of the effects of these variables on birth outcomes. Previous research findings showed an association between maternal nutritional status and birth outcomes, albeit the outcome was mainly on birth weight. The current study extends knowledge in this area by determining the relationship of various maternal nutritional indices with birth length and birth head circumference in addition to birth weight. Gestational weight gain at 26 to 29 weeks was positively associated with infant’s birth weight and birth weight-for-age z-score as well as birth length and birth length-for-age z-score. This finding suggests that low birth length could be a sensitive birth outcome parameter that is associated with inadequate gestational weight gain in a population group with high prevalence of suboptimal maternal nutritional status. Further research is warranted to confirm this finding.

This study has limitations. First, we did not measure the mother’s weight at delivery, hence the full gestational weight gain could not be determined. Previous studies examining the association between total gestational weight gain and birth outcomes reported similar findings [21, 23, 27, 28], and a recent study reported that maternal weight gain during early pregnancy (≤ 20 weeks) and mid pregnancy (21–29 weeks) had a stronger influence on fetal growth and birth outcomes compared to weight gain in later pregnancy (≥ 30 weeks) [24]. Second, using recall pre-pregnancy weight for calculating pre-pregnancy BMI may be subject to recall biases. Third, we used the earliest weight measure in the first trimester as a proxy for pre-pregnancy weight in those subjects (5.2%) who could not recall pre-pregnancy weight. This may underestimate the prevalence of underweight in pregnant women. Finally, the current study was the secondary analysis utilizing the baseline data from a randomized controlled trial that was designed to evaluate the impact of an improved perinatal nutrition care program on breastfeeding, birth and growth outcomes. Hence, the sample size was relatively small, which may limit the generalizability of the results. Future research with larger sample size and wider population groups is warranted to elucidate the underlying mechanisms and the potential roles for maternal nutrition care in optimizing pregnancy outcomes.

## Conclusions

Findings from our study showed that nutrient inadequacies during pregnancy are prevalent among Vietnamese women, often occurring as concurrent inadequacies of multiple nutrients. Maternal underweight prior to pregnancy and women not meeting recommended gestational weight gain were also frequent in our Vietnamese study population.

Our results underscore the need for building policies and implementing public health initiatives aimed to improve dietary quality among girls and women of reproductive age. In particular, prenatal nutrition care must promote optimal gestational weight gain among pregnant women. Such steps are necessary to improve both short- and long-term health outcomes for mothers and their children.

## Data Availability

The data that support the findings of this study are available from Abbott Nutrition, but restrictions apply to the availability of these data, which were used under license for the current study, and so are not publicly available. Data are however available from the authors upon reasonable request and with permission of Abbott Nutrition.

## Abbreviations

BMI: Body mass index
CI: Confidence interval
IOM: Institute of Medicine
WHO: World Health Organization
